# Primary renal osteosarcoma: A case report and review of literature

**DOI:** 10.1002/ccr3.5957

**Published:** 2022-06-15

**Authors:** Farshad Namdari, Mohammad Javad Nazar Pour, Arya Behzadi, Mona Akbari, Hedieh Moradi, Hossein Dialameh

**Affiliations:** ^1^ Aja University of Medical Sciences Imam Reza Hospital Tehran Iran; ^2^ Tehran University of Medical Sciences Sina Hospital Tehran Iran; ^3^ Shahid Beheshti University of Medical Sciences Shohada‐e‐Tajrish Hospital Tehran Iran; ^4^ Iran University of Medical Sciences Pasteur No Hospital Tehran Iran; ^5^ Tehran University of Medical Sciences Sina Hospital, Urology Research Center Tehran Iran

**Keywords:** extra‐osseous osteosarcoma, kidney, primary osteosarcoma, renal osteosarcoma, surgical resection

## Abstract

Primary renal osteosarcoma is an exceedingly rare tumor with poor prognosis. It tends to spread to distant sites and has a low survival rate. In this report, we present a case of a 43‐year‐old female patient presented with flank pain, gross hematuria, weakness, and weight loss. The patient was diagnosed with primary renal osteogenic sarcoma. This case report aimed to describe the clinical and pathological features, pathogenesis, and treatment modalities of this neoplasm.

## Graphical abstract

Primary renal osteosarcoma is a rare tumor with a poor prognosis. Survival of patients is related to the tumor stage and multimodal therapy. The use of genetic testing in high‐risk individuals helps diagnose the tumor at early stages by following up on patients with imaging techniques and improve survival.

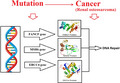

## INTRODUCTION

Primary renal osteosarcoma is an exceedingly rare malignant mesenchymal tumor. There are only 28 cases reported in the literature up to date.^1^ Metastatic skeletal osteosarcoma to the kidney is more common than the primary one.^2^ In addition to the kidneys, extra‐osseous osteosarcoma affects other rare locations such as the brain, lungs, and heart.^3^ It is made of malignant osteoblastic cells producing bone or cartilage. It is usually diagnosed at an advanced stage and about 32% of patients present with metastasis;^4^ therefore, there is a poor prognosis. Treatment modalities such as surgery, chemotherapy, and radiotherapy have palliative effects on this tumor.^5^ Diagnosis of primary renal osteosarcoma is based on the radiologic and histologic findings.^6^ In this article, we present a case of primary osteosarcoma of the kidney and review its clinical presentation, diagnosis, and treatment options.

## CASE REPORT

**FIGURE 1:**
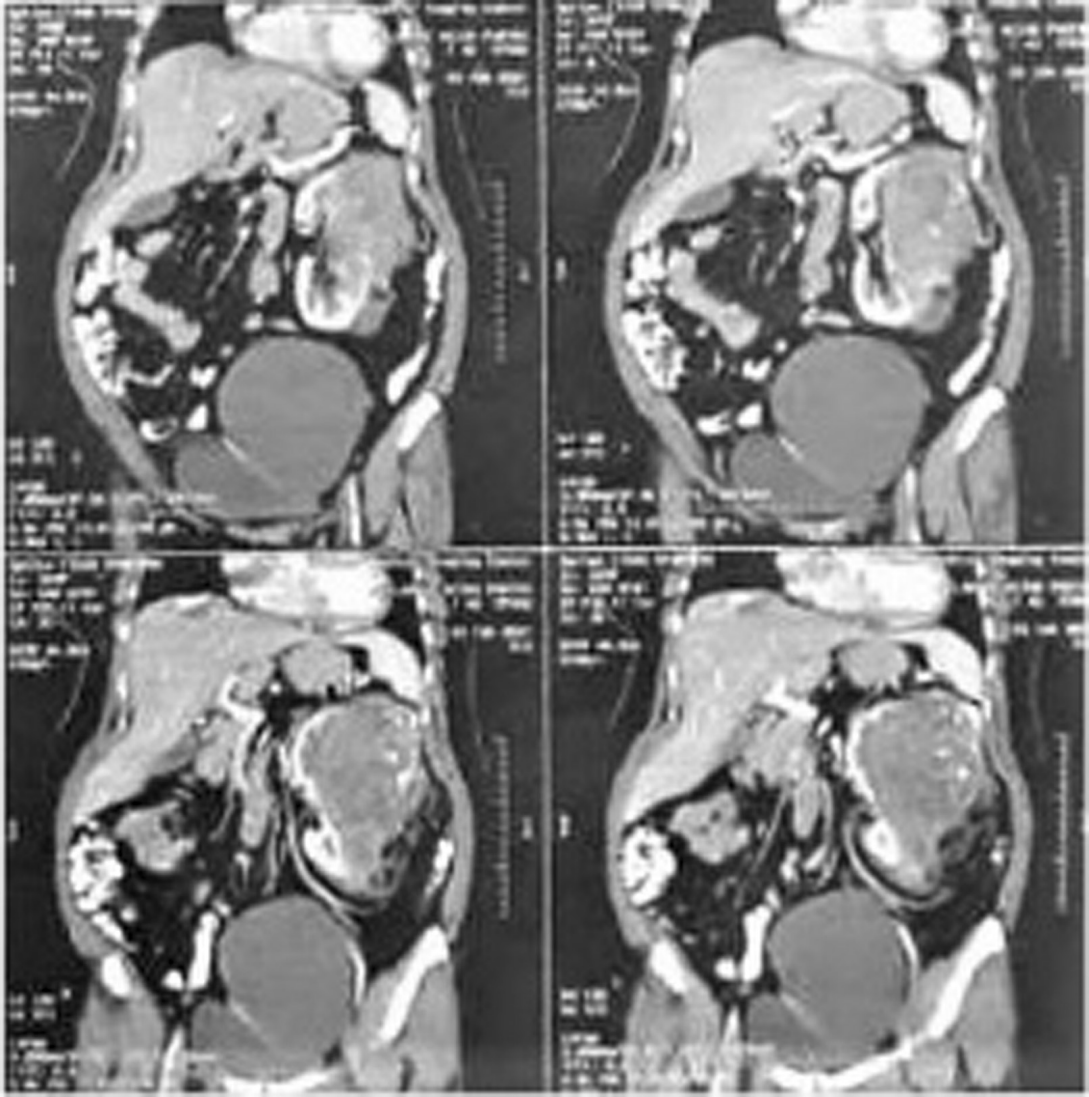
CT Urography showed a large mass originating from the kidney

**FIGURE 2:**
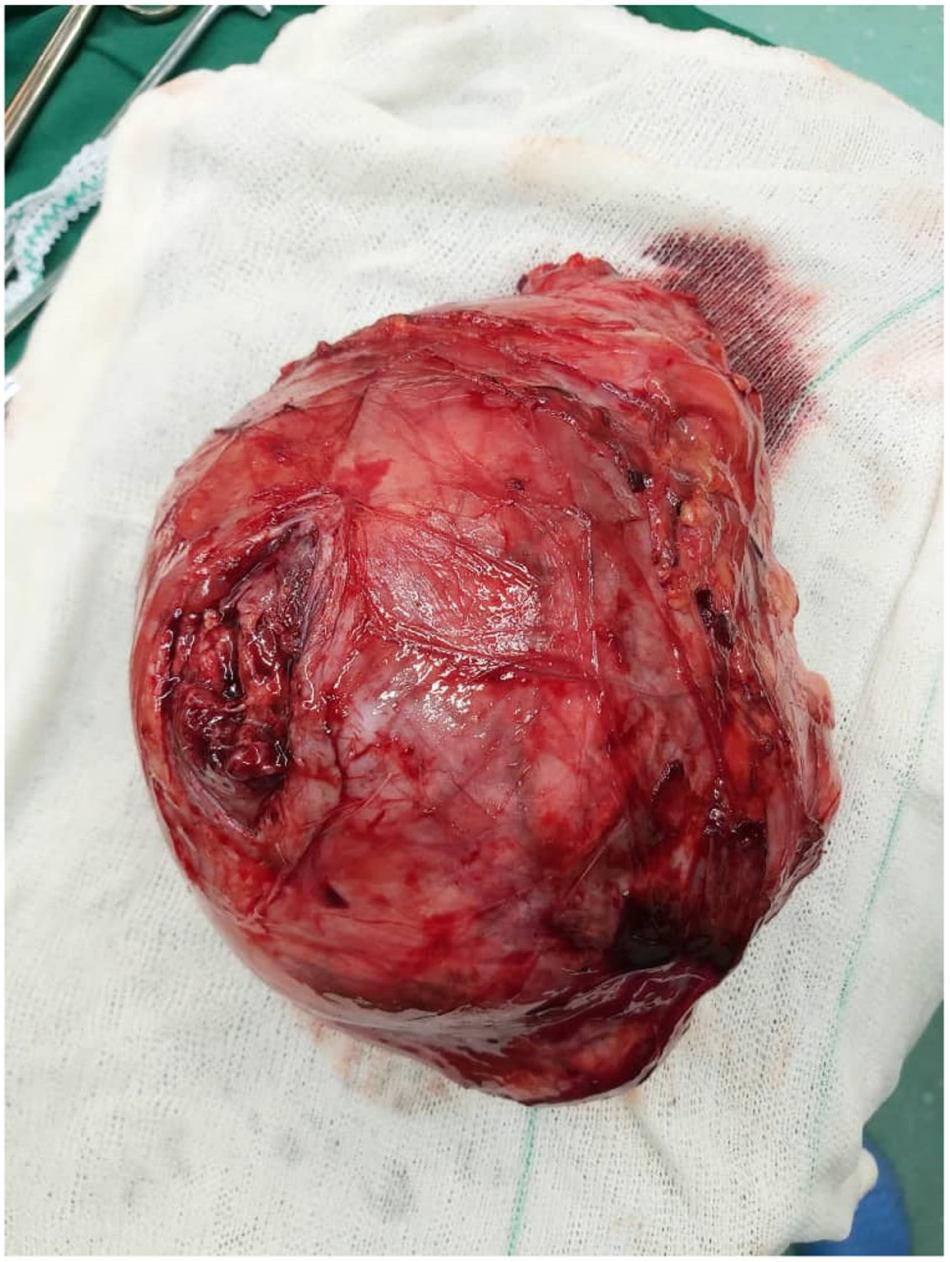
Radical nephrectomy was performed and mass was resected completely

**FIGURE 3:**
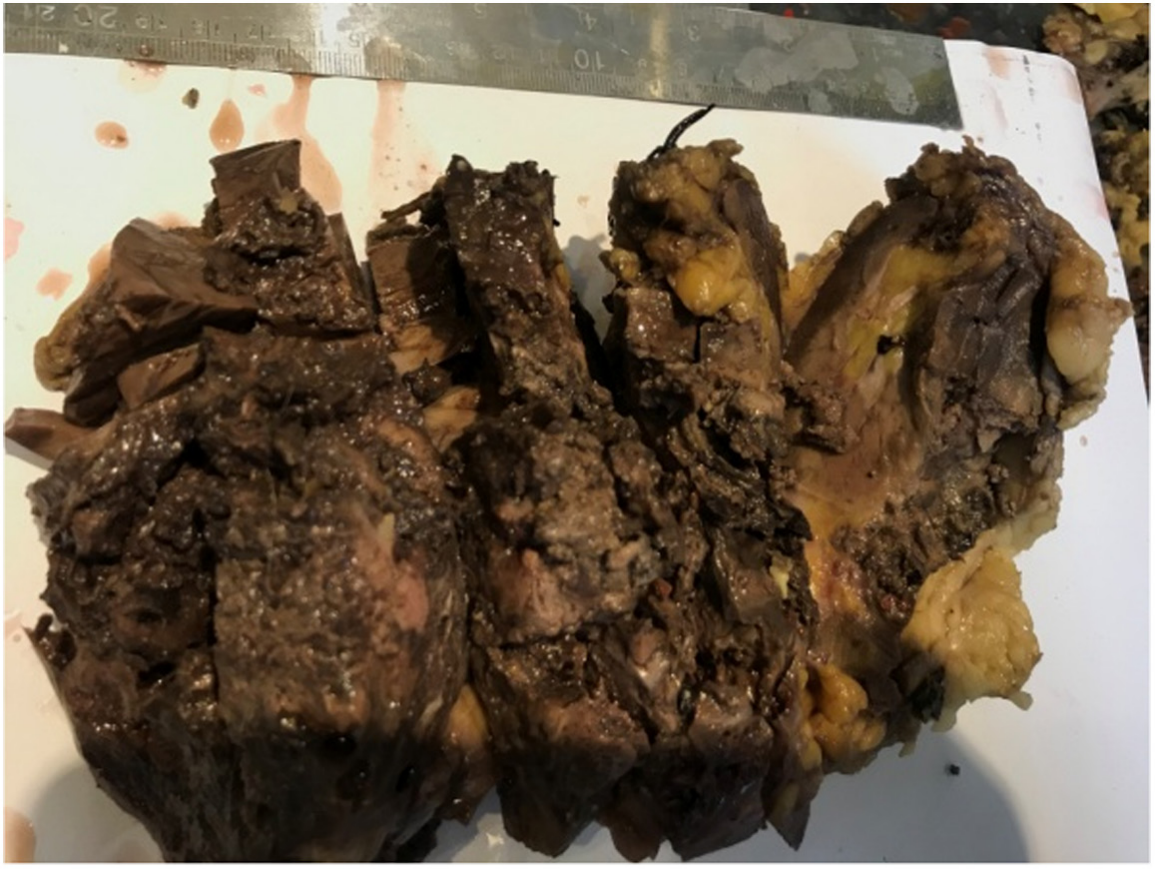
Central yellow‐brownish and rock‐hard mass were shown in the macroscopic examination

**FIGURE 4:**
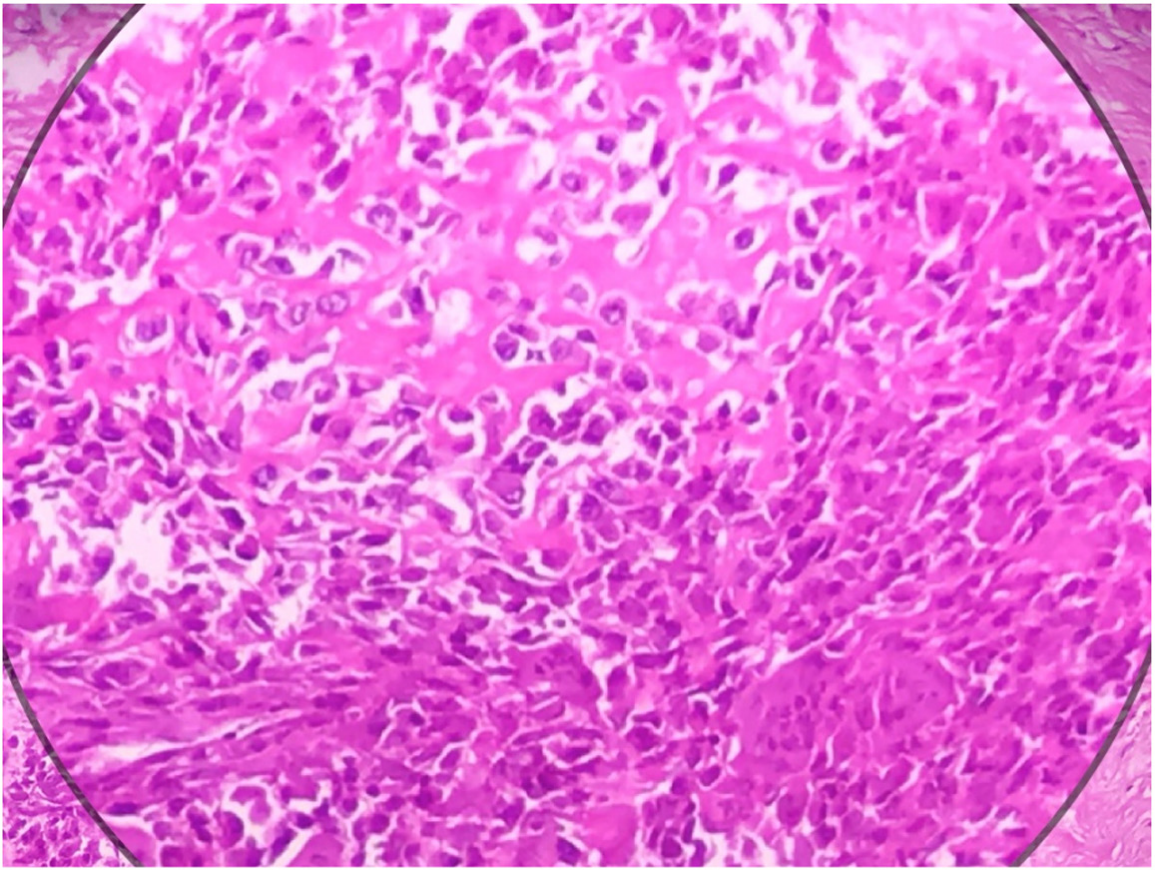
Hematoxylin–eosin (HE) stain (magnification ×100). HE staining shows proliferation of spindle cell with the eosinophilic unmineralized neoplastic osteoid

A 43‐year‐old female patient was admitted with a 3‐month history of flank pain, intermittent gross hematuria, weakness, and weight loss. She had medical history of hypertension. Family history and physical examination were unremarkable. Routine examinations revealed mild anemia (Hb: 11 g/dl) and microscopic hematuria. Urine cytology, blood biochemistry profile, including alkaline phosphatase, liver function test, and stool examination were normal. The patient did not complain of bone pain and neurologic symptoms, as a result, nuclear bone scan and brain imaging were not done. Cystoscopy was also normal. A computed tomography (CT) scan of the abdomen confirmed a large hypodense mass with a maximum diameter of 15 cm in the left retroperitoneal space originating from the kidney. There were irregular foci of calcification (Figure 1). Chest X‐ray revealed no pathologic lesion. Surgical exploration and radical nephrectomy were performed (Figure 2). Macroscopic examination showed a tumor with dimensions of 15 × 10 × 8.5 cm in the kidney and also a surface cut indicated a central yellow‐brownish, hard mass with areas of cystic changes, hemorrhage, necrosis, and prominent calcified whitish areas of firm stone‐like appearance. The tumor invaded the renal sinus, pelvis, and renal sinus fat grossly (Figure 3). Sections in microscopic examination showed a diffuse arrangement of highly cellular tumor having different sizes of spindle cell proliferation with interspersed, unmineralized, neoplastic osteoid. It was associated with high atypical malignant cartilage component and variably pleomorphic tumor cells, intermingled with lace‐like to compact trabeculae. Proliferating cells showed epithelioid features with moderate atypical and a few mitotic features (Figure 4). Surgical margins were tumor‐free and lymphovascular invasion was not identified. She was at a pathologic stage of III (T3aN0M0). Immunohistochemical study was positive for vimentin and CD10 and was negative for Pan CK. The postoperative period in the hospital was uneventful, and the patient had no complications. She was discharged 5 days later. The patient was referred to the oncologic department and received adjuvant chemotherapy (ifosfamide and cisplatinum) and radiotherapy (50 Gy) to the tumor bed. After 3 months and then every 6 months, the patient was followed with chest X‐ray and abdominopelvic CT scan with IV contrast. There was no evidence of local recurrence and distant metastasis during 20 months of follow‐up.

## DISCUSSION

Sarcomas occur in only 1% of renal malignancy with poorer prognosis than other renal cancers. The most common subtype of renal sarcoma is leiomyosarcoma.^7^, ^8^, ^9^ Osteogenic sarcoma originating from the kidney is a rare sarcoma characterized by calcium deposition^10^ with an equal male to female ratio.^11^, ^12^ Symptoms such as flank pain, weight loss, weakness, and gross hematuria were reported in patients with this renal tumor as were seen in the above‐mentioned case.^8^, ^11^, ^13^, ^14^, ^15^, ^16^, ^17^ Its calcified appearance on plain films must be differentiated from calculi, vascular disease, traumatic lesion, cystic lesion.^12^, ^16^ A Sunburst‐like appearance within renal contours on a CT scan is a characteristic radiologic finding.^6^ Microscopic examinations reveal spindle cell proliferation, osteoid production, and the absence of epithelial differentiation. The sarcomatoid variant of renal cell carcinoma (RCC) is a differential diagnosis of renal osteosarcoma and the presence of epithelial cell in histology is an indicator of the sarcomatoid variant of RCC.^16^, ^18^ Pleomorphic osteosarcoma is the most reported subtype and the osteoblastic and the chondroblastic are the rare ones.^19^ Immunohistochemical analysis of primary renal osteosarcoma is positive of vimentin and muscle‐specific actin.^18^ The data reported in the literature and genetic tests performed in these patients, revealed the variations of three genes; MSH6, FANCF, and ERCC4 that as a result of these variations, protein products from these genes will have no function.^4^ Due to the fact that this tumor gets diagnosed at an advanced stage,^20^ it has a poor prognosis with a mortality rate of 80.5%^21^ to 87.5%.^22^

Multimodal treatments, such as surgical resection, radiotherapy, and chemotherapy, are used for this tumor. The choice of treatment in low stage organ‐confined tumors might be surgery alone.^19^ Based on the literature, these treatments did not have promising results.^23^, ^24^, ^25^ Local recurrence and metastasis extending to the peritoneum, lung, liver, and bone marrow are frequent^11^, ^20^, ^26^, ^27^ and early diagnosis is very important in disease control because the aggressiveness of this tumor is related to tumor stage.^19^ maybe the use of genetic testing in high‐risk individuals help diagnose the tumor at early stages by following up on patients with imaging techniques but more studies are needed.

## CONCLUSION

Primary renal osteosarcoma is a rare and fatal tumor with poor prognosis. The survival rate of patients diagnosed with this tumor is related to the tumor stage during diagnosis and multimodal therapy. The use of genetic testing in high‐risk individuals and following them up with imaging techniques will perhaps help to diagnose the tumor at earlier stages. However, further research is needed.

## AUTHOR CONTRIBUTIONS

Study conception and design: H.D; data collection: F.N, H.D; analysis and interpretation of results: M.N, H.M; draft manuscript preparation: M.A, A.B. All authors reviewed the results and approved the final version of the manuscript.

## CONFLICTS OF INTEREST

None.

## CONSENT

Written informed consent was obtained from the patient to publish her clinical and radiological data in accordance with the journal's patient consent policy.

## Data Availability

The data that support the findings of this study are available from the corresponding author upon reasonable request.
